# Neutralizing Antibody Response Dynamics in COVID-19: Insights from Healthy Vaccinees, Breakthrough Infections, and Critically Ill Patients

**DOI:** 10.3390/tropicalmed11070178

**Published:** 2026-06-27

**Authors:** Naveed Ahmed, Wardah Yusof, Nurfadhlina Musa, Kueh Yee Cheng, Nurzulaikha Abdullah, Rosline Hassan, Muhammad Nashrul Farhan Samsudin, Alwi Muhd Besari Hashim, Manickam Ravichandran, Chua Wei Chuan, Chan Yean Yean

**Affiliations:** 1 Department of Medical Microbiology and Parasitology, School of Medical Sciences, Universiti Sains Malaysia, Kubang Kerian 16150, Kelantan, Malaysia; nahmed@ut.edu.sa (N.A.); wardahyusofyaacob@gmail.com (W.Y.); muhammadnashrul@usm.my (M.N.F.S.); weichuan@usm.my (C.W.C.); 2 Department of Emergency Medicine, School of Medical Sciences, Universiti Sains Malaysia, Kubang Kerian 16150, Kelantan, Malaysia; 3 Department of Assistance Medical Science, Applied College, University of Tabuk, Tabuk 71491, Saudi Arabia; 4 Human Genome Centre, School of Medical Sciences, Universiti Sains Malaysia, Kubang Kerian 16150, Kelantan, Malaysia; fadhlina@usm.my; 5 Biostatistics & Research Methodology Unit, School of Medical Sciences, Universiti Sains Malaysia, Kubang Kerian 16150, Kelantan, Malaysia; yckueh@usm.my (K.Y.C.); ngahpc@yahoo.com (N.A.); 6 Department of Hematology, School of Medical Sciences, Universiti Sains Malaysia, Kubang Kerian 16150, Kelantan, Malaysia; roslin@usm.my; 7 Hospital Universiti Sains Malaysia, Universiti Sains Malaysia, Kubang Kerian 16150, Kelantan, Malaysia; 8 Department of Internal Medicine, School of Medical Sciences, Universiti Sains Malaysia, Kubang Kerian 16150, Kelantan, Malaysia; dralwi@usm.my; 9 Faculty of Applied Sciences, Department of Biotechnology, AIMST University, Bedong 08100, Kedah, Malaysia; ravichandran@aimst.edu.my; 10 MyGenome, ALPS Global Holding Berhad, Kuala Lumpur 50400, Malaysia

**Keywords:** COVID-19, neutralizing antibodies, antibody response, vaccination, breakthrough infections, critical illness

## Abstract

Neutralizing antibodies (NABs) play a critical role in assessing the immune response elicited by vaccines, providing insight into their protective efficacy. Despite their importance, there is a notable gap in research directly comparing NAB levels among individuals vaccinated with different vaccines, across diverse ethnicities, and between genders. The study aimed to compare NAB levels across variables such as vaccination status, vaccine type, age, gender, and ethnicity. The NAB levels among different study groups were measured using the Finecare RBD Antibody Test and the cPass kit (ELISA). The data was analyzed statistically using SPSS version 27. A total of 172 study subjects were analyzed. The mean age of participants was 45.03 ± 16.72 years, with 50.9% male and 77.3% of Malay ethnicity. Median NAB levels, assessed by both assays, were highest in females, vaccinated healthy participants, and those who received the Pfizer vaccine. Age-group comparisons revealed variations in median NAB levels across studied groups. Participants aged 10–25 years in the vaccinated healthy (VH) group exhibited the highest median antibody levels; on the other hand, the 26–45 year age group showed the highest median levels in the breakthrough infection (BI) and certain non-vaccinated categories. Ethnic group comparisons highlighted that Malays consistently had the highest median NAB levels. Significant differences in antibody levels were found across vaccination status, ethnicity, and vaccine type (*p* < 0.001). This study underscores the influence of vaccination status, demographic factors, and vaccine type on NAB levels. The Finecare RBD Antibody Test and cPass kit demonstrated comparable trends, highlighting their utility in evaluating vaccine-induced immunity. The findings of this study highlight the need for tailored immunization strategies to optimize protective immunity.

## 1. Introduction

Neutralizing antibodies (NABs) are a specific subset of antibodies that play a critical role in the immune system’s defense against pathogens, particularly viruses [1]. Unlike binding antibodies, which simply attach to a pathogen, neutralizing antibodies can directly block the virus from entering host cells by targeting key viral proteins involved in infection [2]. This prevents the virus from replicating and spreading within the body. The NAB are often generated as part of the body’s adaptive immune response following natural infection or vaccination. Their effectiveness depends on their ability to recognize and bind to viral components such as the spike protein in coronaviruses, thereby neutralizing their infectivity [3,4].

In the context of COVID-19, NABs play a pivotal role in providing immunity against the SARS-CoV-2 virus [5]. Vaccines, particularly those targeting the spike protein, aim to elicit a robust production of these antibodies, which can prevent the virus from binding to the ACE2 receptor on human cells [6]. Studies have shown that vaccinated individuals typically develop strong NAB responses, which provide significant protection against severe disease and hospitalization [4,7]. In cases of breakthrough infections, NABs elicited by prior vaccination or infection can play a critical role in attenuating disease severity and lowering the risk of adverse clinical outcomes. [8]. In individuals who were not vaccinated but became infected, the production of neutralizing antibodies is often associated with the clearance of the virus, though the response may vary based on the severity of the infection and individual immune factors [9,10].

Rapid antibody tests typically detect IgM and IgG antibodies, which are produced by the immune system in response to any infection or disease [11]. IgM is typically the first antibody produced in response to an infection, indicating a recent or acute phase, whereas IgG appears later (in a few weeks and peaks at 6–8 weeks post-immunizing event) and often signifies past exposure or long-term immunity [12]. These rapid tests, often lateral flow immunoassays (LFIAs), work by capturing antibodies from a blood sample using antigen-coated strips, resulting in a color change if the target antibodies are present. They are commonly used for quick, point-of-care detection [13,14].

Different antibody detection methods are based on various mechanisms. Enzyme-Linked Immunosorbent Assay (ELISA) is a widely used technique that works by binding specific antibodies to antigens on a plate, followed by enzyme-linked detection for a colorimetric signal. Chemiluminescent Immunoassay (CLIA) relies on a similar antigen–antibody interaction but uses chemiluminescent labels to produce light as a detection signal [15]. Neutralization assays, on the other hand, directly assess the functional ability of antibodies to block virus entry into cells, rather than just detecting their presence. Each method varies in sensitivity, specificity, and the type of information provided about the immune response [16]. The cPass™ SARS-CoV-2 NAB detection kit (GeneScript, USA) is a Food and Drug Administration (FDA)-approved SARS-CoV-2 kit [17], which uses a competitive ELISA to detect NABs. It is a serological assay that uses a blocking ELISA test to detect functional immunoglobulins that neutralize the interaction between RBD and human angiotensin-converting enzyme 2 (hACE2). The kit measures antibodies’ ability to block the interaction between the SARS-CoV-2 spike protein and the ACE2 receptor, indicating the presence of NABs [18]. cPass assesses the functional activity of the NABs instead of just their presence. While the cPass directly measures NABs by mimicking the virus–host interaction, the Finecare™ RBD Antibody Test detects total antibodies against the RBD of the spike protein, which includes but is not limited to neutralizing antibodies. Although there is a well-documented correlation between anti-RBD antibody levels and neutralizing capacity, not all anti-RBD antibodies confer functional neutralization. The Finecare™ 2019-nCoV RBD Antibody Test (Wondfo, Guangzhou, China) is a rapid lateral flow fluorescence immunoassay, used for qualitative and semi-quantitative detection of spike RBD antibodies of SARS-CoV-2 Spike protein. When the specimen is added to the sample well of the test cartridge, the fluorescence-labeled detector 2019-nCoV RBD protein binds to RBD antibodies in the blood specimen and forms immune complexes [19]. As the complexes migrate on the nitrocellulose membrane by capillary action, the 2019-nCoV RBD antibodies can be captured by another RBD protein that has been immobilized on the test strip. Thus, the more the 2019-nCoV RBD antibody in blood, the higher the signal value scanned by Finecare™ FIA Meters, the stronger the positive degree of the specimen. The default results unit of this test is displayed as relative fluorescence unit (RFU, AU/mL) from Finecare™ FIA meters [19]. Despite the importance of NABs, there is a notable gap in research directly comparing their levels among individuals vaccinated with different vaccines, across diverse ethnicities, and between genders. In the current post-pandemic landscape, understanding the durability and variability of immune responses following COVID-19 vaccination and natural infection remains critically important. As new variants continue to emerge and global vaccination strategies evolve, assessing the comparative effectiveness of different vaccines and the immune profiles of various population groups. Such variability may have implications for vaccine efficacy and public health strategies yet remains underexplored. Hence, this study provides valuable insights into NAB levels across different group of study participants with specific objectives to (1) describe the level of NAB detected by Finecare™ 2019-nCoV RBD Antibody Test (Finecare^TM^) and cPass™ SARS-CoV-2 neutralization antibody detection kit (cPass^TM^) according to type, gender, and ethnicity, and (2) to compare the levels of neutralizing antibody between comparison group detected from Finecare^TM^ and cPass^TM^ detection kit.

## 2. Materials and Methods

### 2.1. Ethical Clearance

This evaluation study was conducted in Hospital Universiti Sains Malaysia (HUSM), Kelantan, Malaysia, from 13 August to 20 September 2021. This study was conducted under the ethical approval of the human research ethics committee of USM (JEPeM) (Ethical approval no. USM/JEPeM/COVID19-44). Written informed consent form was obtained from all subjects involved in the study for their participation in this study and agreement to publish this paper.

### 2.2. Study Subject Categorization and Sample Collection

Peripheral blood was collected from 172 study participants categorized into five groups. The groups are as follows: (1) Vaccinated Healthy (VH): individuals who received a complete COVID-19 vaccination regimen, including at least one booster dose, and did not report any history of SARS-CoV-2 infection. (2) Breakthrough Infection (BI): individuals who were fully vaccinated and subsequently developed a confirmed SARS-CoV-2 infection. (3) Non-Vaccinated (NV): individuals who had not received any dose of a COVID-19 vaccine and had no documented history of SARS-CoV-2 infection. (4) Non-vaccinated with mild infection (NVC1): non-vaccinated individuals who experienced a confirmed SARS-CoV-2 infection with mild symptoms (categorized as WHO clinical classification category 1–2). (5) Non-vaccinated with moderate to severe infection (NVC2): non-vaccinated individuals who experienced a confirmed SARS-CoV-2 infection with moderate to severe symptoms (categorized as WHO clinical classification category 3–5). Confirmed COVID-19 patients were classified based on the signs and symptoms of the disease as stated in Annex 2e: Clinical Management of Confirmed COVID-19 Cases in Adults and Paediatric by the Malaysia Ministry of Health (MOH, 2020) [20].

Participants (VH and BI) in this study received one of the following COVID-19 vaccines as part of their full vaccination regimen, including at least one booster dose: (1) Sinovac–CoronaVac (inactivated virus vaccine, Beijing, China), (2) Pfizer–BioNTech (mRNA-based vaccine, Mainz, Germany), and (3) Oxford–AstraZeneca (viral vector-based vaccine, Cambridge, United Kingdom). All vaccinated individuals received their primary two-dose series followed by a booster dose, administered according to national immunization guidelines, with dosing intervals ranging from 4 to 12 weeks between the primary doses and a minimum of 3 months before the booster dose. All participants’ vaccination and infection status were initially verified through official medical records provided by the hospital, including vaccination certificates and documented RT-PCR results for COVID-19 infection.

Participants were categorized into age groups (10–25, 26–45, and ≥46 years) to reflect known differences in immune response. This stratification considers the working-age population and age-related immunosenescence and has been applied in previous vaccine response studies [21,22].

### 2.3. Finecare RBD Antibody Test (Wondfo RBD/Neutralizing Antibody)

The detection of anti-RBD binding antibody levels was carried out using the Finecare RBD Antibody Test (Wondfo, Guangzhou, China). Twenty microliters (20 µL) of whole blood was added to the detection buffer supplied by the kit and mixed thoroughly by inverting the tube. Then, 75 µL of the mixture was loaded into the test cassette and left at room temperature for 15 min before the test cassette was inserted into the Finecare™ FIA Meters (Wondfo, Guangzhou, China) machine for antibody detection reading. The result was displayed as relative fluorescence units (RFU, AU/mL), which can be converted to WHO binding antibody units (BAU)/mL, where 1 AU/mL is equal to 20 BAU/mL. A reading of ≥1 AU/mL or 20 BAU/mL indicates positive antibody detection [23].

### 2.4. cPass™ SARS-CoV-2 Neutralization Antibody Detection Kit (GenScript, Piscataway, USA)

For peripheral (capillary) blood plasma collection, whole peripheral blood was pipetted into a sterile 1.5 mL microfuge tube using a 200 µL pipette and centrifuged at 1000× *g* for 10 min to pellet the cells. The top plasma layer was carefully transferred into a fresh microfuge tube, avoiding the pellet. Plasma may be used immediately for the cPass assay or stored at 4 °C for long-term storage. The detection of NABs for all samples was carried out according to the manufacturer’s recommendations [24]. A standard curve was used to plot the neutralization response in the given samples. The final neutralization titer was calculated for each sample to help quantify the neutralization activity. The results identified samples with low (<1500 U/mL), medium (1500–5000 U/mL), and high (>5000 U/mL) neutralization titers.

### 2.5. Statistical Analysis

All data analysis was done using IBM SPSS Statistics for Windows, Version 27.0 (IBM Corp., Armonk, NY, USA). The demographic characteristics of respondents were described using descriptive statistics by reporting the mean and standard deviation (SD) for the numerical variable (age). Continuous variables were summarized as mean ± SD, while categorical variables were presented as frequencies and percentages (ethnic, gender, comorbid, et cetera).

The distribution of continuous variables was assessed visually using histograms and box-and-whisker plots and statistically using the Kolmogorov–Smirnov and Shapiro–Wilk tests. For normally distributed data, comparisons between two groups were performed using the independent-samples *t*-test, while comparisons among more than two groups were performed using one-way analysis of variance (ANOVA) with Tukey’s post hoc test. For non-normally distributed data, comparisons between two groups were performed using the Mann–Whitney test, while comparisons among more than two groups were performed using the Kruskal–Wallis test followed by pairwise post hoc comparisons using the Mann–Whitney test.

To account for multiple pairwise comparisons, the Bonferroni correction was applied where appropriate. For analyses involving 10 pairwise comparisons, the adjusted significance threshold was set at *p* < 0.005 (0.05/10). The association between categorical variables was assessed using Pearson’s chi-square (χ^2^) test. To evaluate the relationship between the Finecare RBD Antibody Test and cPass assay results, Spearman’s rank correlation analysis was performed. Unless otherwise specified, statistical significance was defined as a two-tailed *p* < 0.05.

## 3. Results

### 3.1. Participant Characteristics

A total of 172 samples were included in this study. The data obtained were expressed as mean with SD for numerical variables and frequency (*n*) with percentage (%) for categorical variables, as tabulated in Table 1. The mean age of all participants was 45.03 years, with a standard deviation of 16.72. More than half of the samples were obtained from males (*n* = 87, 50.9%) individuals comparing to the females. The majority of the participants were Malay ethnic, comprising 77.3% of the total (*n* = 133).

### 3.2. Level of Finecare RBD Antibody Test and cPass Kit Between Different Groups of Variables

**Figure 1:**
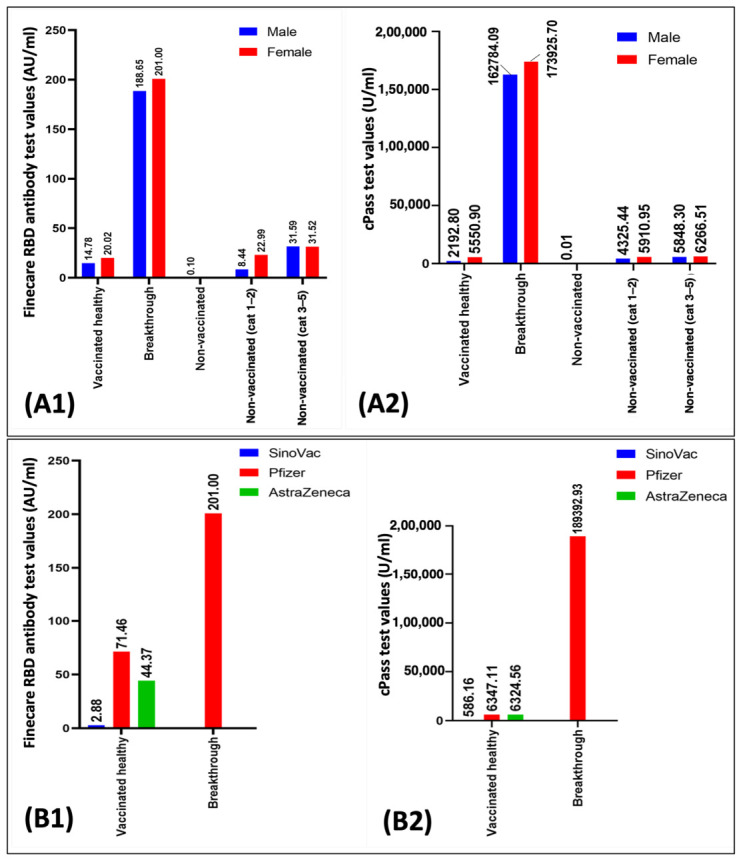
Distribution of median data. (**A1**) Finecare RBD Antibody Test by gender, (**A2**) cPass Kit values by gender. (**B1**) Finecare RBD Antibody Test by types of vaccines, (**B2**) cPass Kit values by types of vaccines.

The distribution of antibody responses, as measured by both the Finecare RBD Antibody Test and the cPass Kit, revealed notable differences across different variables among the different tested groups. Figure 1 illustrates trends in median antibody levels across different variables. Higher median antibody levels were observed among female participants compared with male participants across the study groups. Nevertheless, these differences did not reach statistical significance and therefore cannot be interpreted as evidence of a sex-related effect on antibody responses. Among vaccine types, the Pfizer–BioNTech vaccine generated the strongest antibody responses in the VH group. The individuals aged 10–25 years had the highest antibody levels in the VH group, while the 26–45 age group showed peak responses in BI and NVC2 categories. Ethnically, Malay participants had the strongest median responses in both assay systems, suggesting possible host-related variations in immune response.

**Figure 2:**
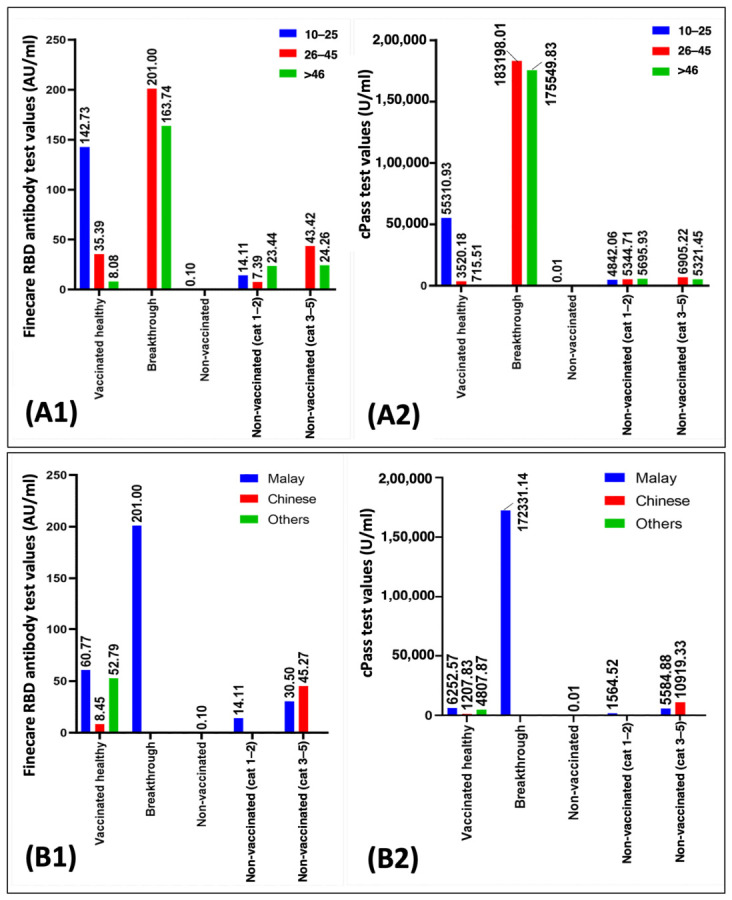
Distribution of median data. (**A1**) Finecare RBD Antibody Test by age group, (**A2**) cPass Kit by age group. (**B1**) Finecare RBD Antibody Test by ethnicity, (**B2**) cPass Kit values by ethnicity.

Figure 1(A1,A2) compares the Finecare RBD Antibody Test and cPass Kit values between genders for each category of vaccination status. Female participants demonstrated higher median Finecare RBD antibody and cPass NAB levels across vaccination categories; however, no statistically significant sex-related differences were observed. Figure 1(B1,B2) below compares the Finecare RBD Antibody Test and cPass Kit neutralization antibodies between different vaccine types for each vaccination status category. The highest median was for the Pfizer Vaccine in the Vaccinated Healthy group. Figure 2(A1,A2) compares Finecare RBD Antibody Test and cPass Kit values for each vaccination status category between different age groups. In the VH group, the highest median Finecare RBD antibody and cPass NAB levels were observed among participants aged 10–25 years. In NVC1, the highest median values were observed in participants aged ≥46 years, whereas in the BI and NVC2, the highest median values were observed in the 26–45-year age group. Figure 2(B1,B2) compares the median Finecare RBD Antibody Test and cPass Kit value between different ethnic groups for each category of vaccination status. The highest median values were found in the Malay group for both assays. A strong positive correlation was observed between Finecare and cPass measurements (Spearman’s ρ = 0.78, *p* < 0.001).

### 3.3. Neutralization Antibody Detected from Finecare RBD Antibody Test Between Groups of Different Variables

The comparison of NAB levels detected from the Finecare RBD Antibody Test between different tested groups has been summarized in Table 2. Based on the result, there were significant differences for variables of the status of the vaccination group (*p* < 0.001) and vaccination type (*p* < 0.001). Individuals with BIs exhibited the highest median antibody levels, followed by those with NVC2. The VH individuals had lower levels compared to BI cases, but were still significantly higher than non-vaccinated groups. Differences by vaccine type were also substantial, with Pfizer recipients showing the highest median values, followed by Oxford–AstraZeneca and Sinovac–CoronaVac.

Pairwise comparisons (Table 3) demonstrated that participants with BI exhibited significantly higher antibody levels than VH individuals and all NV categories. The significant pairs were (1) VH vs. BI (*p* < 0.001), (2) VH vs. NV (*p* < 0.001), (3) BI vs. NV (*p* = 0.001), (4) BI vs. NVC1 (*p* < 0.001), (5) BI vs. NVC2 (*p* < 0.001), and (6) NV vs. NVC2 (*p* = 0.004). No statistically significant differences were observed between VH participants and the NVC1/NVC2 groups.

### 3.4. Comparison of cPass Kit Neutralization Antibody Levels Between Groups of Different Variables

The comparison of the cPass Kit circulating neutralizing antibodies between the groups of different variables was summarized in Table 4, supporting similar trends as seen with Finecare.

Breakthrough cases had the highest NAB levels, while non-vaccinated individuals showed minimal response. Notably, Malay participants again showed higher antibody levels compared to other ethnicities (*p* < 0.001), and Pfizer–BioNTech recipients exhibited the strongest humoral immune responses (*p* < 0.001) among the vaccine groups.

Table 5 further validated these findings, showing significant differences between BI cases and all other groups. The VH individuals also showed significantly higher NAB levels than non-vaccinated individuals, but not as high as the BI group. Among non-vaccinated groups, those with more severe symptoms tended to have higher antibody levels than those with mild symptoms, though some comparisons were not statistically significant after correction. Seven pairs of the comparison groups were significantly different based on the median levels of the cPass Kit (*p* < 0.005). The significant pairs were (1) VH vs. BI (*p* < 0.001), (2) VH vs. NV (*p* = 0.002), (3) BI vs. NV (*p* = 0.001), (4) BI vs. NVC1 (*p* < 0.001), (5) BI vs. NVC2 (*p* < 0.001), (6) NV vs. NVC1 (*p* = 0.002), and (7) NV vs. NVC2 (*p* = 0.004).

## 4. Discussion

The NABs play an important role in the immune response against infectious agents by preventing the entry and replication of pathogens in host cells. These antibodies are particularly important in the context of viral infections such as COVID-19, where they serve as a key mechanism of immunity elicited by natural infection or vaccination [25,26,27]. Different assays are commonly employed to measure NAB levels, but the comparative reliability, sensitivity, and consistency of these methods are not well-documented [28,29,30]. Establishing a standardized understanding of how these assays perform relative to each other is essential for ensuring the accuracy of immunogenicity assessments across studies and populations. This study was therefore conducted to address these gaps by systematically comparing NAB levels across vaccines, demographic groups, and genders, while also evaluating the concordance between two widely used NAB assays.

The results of the present study demonstrated variations in median antibody levels across age groups. Individuals aged 10–25 years exhibited the highest median antibody levels within the VH category, whereas higher median levels were observed among older age groups in certain NV categories. Although females and Malay participants demonstrated higher median antibody levels in several categories, these observations were not consistently supported by statistically significant differences and should therefore be interpreted with caution. The study population was predominantly composed of participants of Malay ethnicity, with relatively limited representation from other ethnic groups. The Malay ethnicity showed a higher NAB level compared to other ethnicities. However, this might be due to the high number of Malay patients recruited (77%) compared to the Chinese and other ethnicities. This finding should be interpreted cautiously, as it may reflect the demographic composition of the study cohort and recruitment patterns rather than true biological differences in immune responses. The small sample sizes of non-Malay ethnic groups may have reduced the statistical power to detect meaningful inter-ethnic differences and increased susceptibility to sampling variability.

Although antibody levels provide valuable information regarding the immune status of participants at the time of assessment, they may not fully reflect long-term protection, as immune responses evolve dynamically following vaccination and/or natural infection [21]. An important consideration in the interpretation of NAB responses is the timing of sample collection following vaccination. Although the exact interval between vaccination and blood sample collection was not uniformly documented for all participants, available data indicate that the second vaccine doses were administered between March and early September 2021. Based on this timeline, most samples were collected approximately 6 to 8 weeks post-vaccination, which aligns with the period when peak antibody responses are generally expected. This window provides a reasonable estimation of post-vaccination immune response; however, variability in timing may have introduced some degree of heterogeneity in antibody levels, which should be considered when comparing groups. Future studies with more precise timing records may help refine our understanding of the kinetics of NAB production.

The emergence of the Delta variant caused a significant surge in COVID-19 cases worldwide, including among vaccinated individuals, highlighting a concerning rise in breakthrough infections as well as other infections [31]. Studies reported that 19.2% of these infections were attributed to breakthrough cases, raising questions about vaccine-induced immunity against highly transmissible variants like Delta [32]. This situation sparked widespread debate regarding the durability and effectiveness of existing vaccines and whether additional booster doses were necessary to enhance protection. Investigations into NAB levels revealed a potential correlation between waning immunity and the increased susceptibility to Delta-driven infections. The decline in NAB titers over time underscored the need for updated strategies, including booster campaigns, to maintain robust immune defense against evolving variants.

The sensitivity of Finecare^TM^ was lower than the cPass^TM^ assay, as observed in Table 3 and Table 5, in which significant differences between groups were detected in five groups from cPass^TM^ compared to four groups by Finecare^TM^. The results for both assays were also not directly comparable since both assays use different units for results interpretation, with Finecare^TM^ using RFU, AU/mL, or BAU/mL and cPass^TM^ using U/mL. Hence, a direct quantitative comparison between the Finecare and cPass assays should be interpreted with caution because the assays employ different analytical methodologies, calibration systems, and reporting units. In the absence of a validated conversion factor, direct standardization of cPass results to BAU/mL was not feasible. The greater discriminatory capacity observed with the cPass assay may reflect differences in analytical sensitivity and the measurement of functional neutralizing antibodies.

Although the principle of detection for both Finecare and cPass^TM^ differs, both showed that NVC2 exhibited a higher level of NABs than NVC1, showing that a higher titre of NAB correlates with symptom severity. This is supported by a previous study showing that disease severity was independently associated with neutralizing antibody levels [25]. However, the antibody levels for non-vaccinated patients in both severity categories were not significantly different from those of healthy vaccinated patients. Forgacs et al. (2021) reported that serological protection conferred by vaccination was significantly more robust than antibodies induced by natural viral infection [33]. They also reported that vaccination produced higher antibody titres in participants previously infected by SARS-CoV-2 than ‘naive’ participants, indicating memory B cell-mediated immune responses producing robust high-affinity antibodies recalled from prior infection [33].

The progressive decline of vaccine effectiveness (VE) assessed by multiple studies, regardless of the vaccine used, postulated that the VE decline is correlated with an increased risk of developing symptoms during breakthrough infection [34,35]. They also postulated that an inadequate vaccine response producing low antibody levels can serve as a predictive indicator of a potentially higher susceptibility to breakthrough infection.

A comparison between groups VH, BI, NV, NVC1, and NVC2 antibody levels showed that BI patients’ NAB levels for both analyses were significantly higher than those of VH, NV, and NVC1/NVC2 patients. The extremely high level of NABs in the BI group showed that even though the patients were not fully protected, a second infection produced higher NAB excretion compared to the symptomatic non-vaccinated patients from groups NVC1 and NVC2, leading to lower infection severity. This was discussed by Walls et al. (2022) [36], who reported that breakthrough infection or infected-vaccinated (infected before vaccination) patients showed greater breadth, potency, and durability of serum-neutralizing activity than those who were only infected by SARS-CoV-2 or naive individuals who received complete vaccination doses. Another study showed no significant difference in NAB levels was observed among the breakthrough cases who were vaccinated with mRNA or AstraZeneca vaccines, both with prior COVID-19 infection, following the first and six months after the second dose [1].

The data from both analyses showed there was a significant difference in NAB levels detected from patients vaccinated using the three types of vaccines, with the highest level detected from Pfizer–BioNTech. The results showed a significant increase in NAB levels after complete vaccination with all vaccine types. These differences could also be because of the timeline when patients were recruited, different vaccine types, and different modes of action, etc. Similar results were seen from another previous study where those who were vaccinated with Moderna or Pfizer exhibited the highest NAB titers, followed by AstraZeneca, and, finally, Sinovac with the lowest titer [1].

Study Limitations: Certain limitations should be considered when interpreting the findings of this study. First, the cross-sectional design provided an assessment of antibody responses at a single time point, thereby offering only a temporal snapshot of the humoral immune status of the study population. Second, the relatively small sample size of certain comparison groups, particularly the Oxford–AstraZeneca vaccine group (*n* = 3) and the non-vaccinated control group (*n* = 4), may reduce statistical power, increase susceptibility to random variation, and limit the precision and reliability of subgroup-specific estimates. Third, age-related data were unavailable for 26 participants, necessitating the use of available-case analyses for age-stratified comparisons. Although age-related analyses were performed using available case data, the missing information may have reduced the accuracy of age-stratified comparisons and could have introduced bias if the missing data were not randomly distributed. Therefore, age-related findings should be interpreted with appropriate caution. Another limitation of the study was that the gender information was missing for one participant. Although the extent of missing data was minimal and unlikely to have influenced the study findings, this should be considered when interpreting the demographic characteristics of the study population.

## 5. Conclusions

In conclusion, this study demonstrated significant differences in NAB levels across vaccination and infection-status groups as measured by both the Finecare RBD Antibody Test and the cPass assay. Participants with BI exhibited the highest antibody levels and showed significantly greater responses than VH individuals and NV groups. Variations in median antibody levels were observed across age groups, sex, ethnicity, and vaccine types; however, several of these differences were not statistically significant and should therefore be interpreted cautiously. The findings highlight the importance of vaccination and prior infection history in shaping humoral immune responses and support continued monitoring of antibody responses across different population groups. Furthermore, future longitudinal studies incorporating repeated immunological assessments are warranted to characterize the long-term kinetics of antibody responses and their relationship with breakthrough infections and clinical protection.

## Figures and Tables

**Table 1:** Descriptive statistics of all samples (*n* = 172).

Variables		Mean (SD)	*n* (%)
Age (Years) *	10–25	45.03 (16.72)	23 (15.8)
	26–45		53 (36.3)
	≥46		70 (47.9)
Group	VH	-	47 (27.3)
	BI		33 (19.2)
	NV		4 (2.3)
	NVC1		46 (26.7)
	NVC2		42 (24.4)
Gender **	Male	-	87 (50.9)
	Female		84 (49.1)
Ethnicity	Malay	-	133 (77.3)
	Chinese		33 (19.2)
	Others		6 (3.5)
Vaccine type	Sinovac–CoronaVac	-	14 (8.1)
	Pfizer–BioNTech		36 (20.9)
	Oxford–AstraZeneca		3 (1.7)

* Age-group information was available for 146 participants; data were missing for 26 participants. Percentages were calculated based on the number of participants with available age information. ** Gender information was available for 171 participants; data were missing for one participant. Percentages were calculated based on the number of participants with available gender information. VH: Vaccinated Healthy. BI: Breakthrough Infection. NV: Non-Vaccinated. NVC1: Non-vaccinated individuals who experienced a confirmed SARS-CoV-2 infection with mild symptoms (categorized as WHO clinical classification category 1–2). NVC2: Non-vaccinated with moderate to severe infection.

**Table 2:** Comparison of the anti-RBD binding antibody levels detected between groups using the Finecare RBD Antibody Test.

Variables Group		Median (IQR)	Statistic Value	*p*-Value
Status of vaccination	VH	24.01 (79.78)	288.60	<0.001 *
	BI	201.00 (107.06)		
	NV	0.11 (0.12)		
	NVC1	14.11 (38.64)		
	NVC2	31.59 (64.68)		
Gender	Male	24.93 (1.26)	2984.00	0.172 #
	Female	39.01 (123.50)		
Ethnicity	Malay	40.32 (106.82)	5.29	0.071 *
	Chinese	8.45 (57.61)		
	Others	14.78 (111.08)		
Vaccine type	Sinovac–CoronaVac	2.88 (7.07)	25.60	<0.001 *
	Pfizer–BioNTech	96.55 (150.30)		
	Oxford–AstraZeneca	44.37 (0.00)		

* Kruskal–Wallis, # Mann–Whitney, IQR: Interquartile Range. VH: Vaccinated Healthy. BI: Breakthrough Infection. NV: Non-Vaccinated. NVC1: Non-vaccinated individuals who experienced a confirmed SARS-CoV-2 infection with mild symptoms (categorized as WHO clinical classification category 1–2). NVC2: Non-vaccinated with moderate to severe infection.

**Table 3:** Pairwise comparison of anti-RBD binding antibody levels using Finecare RBD Antibody Test between groups.

Variables Group		Statistic Value	*p*-Value
Based on vaccination status	VH vs. BI	276.00	<0.001
	VH vs. NV	1.00	<0.001
	VH vs. NVC1	821.00	0.065
	VH vs. NVC2	823.00	0.675
Based on infection category	BI vs. NV	4.00	0.001
	BI vs. NVC1	143.00	<0.001
	BI vs. NVC2	164.00	<0.001
	NV vs. NVC1	14.50	0.006
	NV vs. NVC2	8.00	0.004
	NVC1 vs. NVC2	673.00	0.137

Bonferroni correction on alpha value (0.05/10), alpha = 0.005. VH: Vaccinated Healthy. BI: Breakthrough Infection. NV: Non-Vaccinated. NVC1: non-vaccinated individuals who experienced a confirmed SARS-CoV-2 infection with mild symptoms (categorized as WHO clinical classification category 1–2). NVC2: non-vaccinated with moderate to severe infection.

**Table 4:** Comparison of circulating neutralizing antibodies using cPass Kit between groups of different variables.

Variables Group		Median (IQR)	Statistic Value	*p*-Value
Status of vaccination	VH	3164.41 (6763.08)	22.32	<0.001 *
	BI	172,331.14 (118,548.34)		
	NV	0.00 (150.86)		
	NVC1	5224.67 (5164.40)		
	NVC2	6529.94 (13,842.86)		
Gender	Male	4962.01 (24,575.38)	2810.50	0.184 #
	Female	6854.88 (13,175.72)		
Ethnicity	Malay	7962.14 (26,419.43)	18.99	<0.001 *
	Chinese	1207.83 (6291.82)		
	Others	2371.37 (18,286.25)		
Vaccine type	Sinovac–CoronaVac	588.85 (1476.01)	22.32	<0.001 *
	Pfizer–BioNTech	17,352.57 (151,255.49)		
	Oxford–AstraZeneca	6324.56 (0.00)		

* Kruskal–Wallis, # Mann–Whitney, IQR: Interquartile Range. VH: Vaccinated Healthy. BI: Breakthrough Infection. NV: Non-Vaccinated. NVC1: non-vaccinated individuals who experienced a confirmed SARS-CoV-2 infection with mild symptoms (categorized as WHO clinical classification category 1–2). NVC2: non-vaccinated with moderate to severe infection.

**Table 5:** Pairwise comparison value of cPass Kit between groups.

Group Variables		Statistic Value	*p*-Value
Based on vaccination status	VH vs. BI	45.00	<0.001
	VH vs. NV	3.00	0.002
	VH vs. NVC1	750.00	0.071
	VH vs. NVC2	609.00	0.141
Based on infection category	BI vs. NV	0.00	0.001
	BI vs. NVC1	42.00	<0.001
	BI vs. NVC2	65.00	<0.001
	NV vs. NVC1	5.00	0.002
	NV vs. NVC2	7.50	0.004
	NVC1 vs. NVC2	775.50	0.624

Bonferroni correction on alpha value (0.05/10), alpha = 0.005. VH: Vaccinated Healthy. BI: Breakthrough Infection. NV: Non-Vaccinated. NVC1: non-vaccinated individuals who experienced a confirmed SARS-CoV-2 infection with mild symptoms (categorized as WHO clinical classification category 1–2). NVC2: non-vaccinated with moderate to severe infection.

## Data Availability

More data will be provided upon a reasonable request to the corresponding author.

## Abbreviations

The following abbreviations are used in this manuscript:

NABs	neutralizing antibodies
LFIAs	lateral flow immunoassays
ELISA	Enzyme-Linked Immunosorbent Assay
CLIA	Chemiluminescent Immunoassay
FDA	Food and Drug Administration
hACE2	human angiotensin-converting enzyme 2
RFU	relative fluorescence unit
HUSM	Hospital Universiti Sains Malaysia
BI	breakthrough infection
BAU	binding antibody units
SD	standard deviation
